# Accelerometer-Measured Sedentary Behavior Patterns, Brain Structure, and Cognitive Function in Dementia-Free Older Adults: A Population-Based Study

**DOI:** 10.3233/JAD-230575

**Published:** 2023-11-07

**Authors:** Xiaolei Han, Lin Song, Yuanjing Li, Yi Dong, Rui Liu, Qi Han, Xiaojie Wang, Ming Mao, Lin Cong, Shi Tang, Tingting Hou, Qinghua Zhang, Cuicui Liu, Xiaodong Han, Lin Shi, Lars Nyberg, Lenore J. Launer, Yongxiang Wang, Yifeng Du, Chengxuan Qiu

**Affiliations:** a Department of Neurology, Shandong Provincial Hospital Affiliated to Shandong First Medical University, Jinan, Shandong, China; b Department of Neurology, Shandong Provincial Hospital, Shandong University, Jinan, Shandong, China; c Institute of Brain Science and Brain-inspired Research, Shandong First Medical University & Shandong Academy of Medical Sciences, Jinan, Shandong, China; d Aging Research Center and Center for Alzheimer Research, Department of Neurobiology, Care Sciences and Society, Karolinska Institutet-Stockholm University, Stockholm, Sweden; e BrainNow Research Institute, Shenzhen, Guangdong, China; f Department of Imaging and Interventional Radiology, The Chinese University of Hong Kong, Hong Kong SAR, China; g Department of Radiation Sciences, Radiology, Umeå University, Umeå, Sweden; h Intramural Research Program, Laboratory of Epidemiology and Population Sciences, National Institute on Aging, National Institutes of Health, Baltimore, MD, USA

**Keywords:** Accelerometer, Alzheimer’s disease, brain aging, cognition, magnetic resonance imaging, population-based study, sedentary behavior patterns

## Abstract

**Background:**

Sedentary behavior is associated with cognitive impairment, but the neuropathological mechanisms underlying their associations are poorly understood.

**Objective:**

To investigate the associations of accelerometer-measured sedentary behavior patterns with brain structure and cognition, and further to explore the potential mechanisms.

**Methods:**

This community-based study included 2,019 older adults (age≥60 years, 59% women) without dementia derived from participants in the baseline examination of MIND-China (2018–2020). We assessed sedentary parameters using an accelerometer and cognitive function using a neuropsychological test battery. Structural brain markers were assessed on the structural brain MRI scans in a subsample (*n* = 1,009). Data were analyzed using the general linear, isotemporal substitution, and mediation models.

**Results:**

In the total sample (*n* = 2,019), adjusting for multiple covariates and moderate-to-vigorous-intensity physical activity, longer mean sedentary bout duration was linearly related with lower *z-*scores of global cognition, verbal fluency, and memory (p_trend_ < 0.05), whereas greater total sedentary time was linearly associated with lower z-scores of global cognition, verbal fluency, and memory only among individuals with long sedentary time (>10 h/day) (p_trend_ < 0.05); Breaking up sedentary time with same amount of light-intensity physical activity was significantly associated with higher verbal fluency and memory *z-*scores (*p* < 0.05). In the MRI subsample (*n* = 1,009), separately entering structural brain MRI markers into the mediation models substantially attenuated the associations of mean sedentary bout duration with global cognition, verbal fluency, and memory *z-*scores.

**Conclusion:**

Prolonged uninterrupted sedentary time is associated with poor global cognition, memory, and verbal fluency among rural older adults, and structural brain markers could partially mediate the association.

## INTRODUCTION

Sedentary behavior is defined as any waking behavior characterized by an energy expenditure≤1.5 metabolic equivalents in a sitting, reclining, or lying posture [1]. Sedentary behavior has been associated with various adverse health outcomes such as obesity, diabetes, cardiovascular disease, and mortality [2, 3]. Systematic reviews supported the association of sedentary behavior with cognitive impairment and dementia [4, 5]. However, most of the previous studies had relied on self-reported sedentary behavior, in which the findings might be affected by recall bias [6, 7].

Previously, accelerometer-measured total sedentary time has been associated with poor cognitive function in some studies [8–10], but not in others [11, 12]. Apart from total sedentary time, patterns of sedentary behavior, such as average duration of prolonged sedentary time (sedentary bouts) and frequency of interrupted sedentary time (sedentary breaks), appear to be related to cognitive outcomes. The randomized controlled trials showed that replacing prolonged sitting with short bouts of light-to-moderate intensity activity could promote postprandial glucose metabolism [13, 14], suggesting that both the total sedentary time and the manner in which it is accumulated are associated with health outcomes. However, population-based studies have rarely explored the association between accruing sitting time in prolonged, uninterrupted bouts and cognitive function, especially among rural-dwelling older adults, who usually have lower education, poorer socioeconomic status, and worse lifestyles compared with urban populations.

The potential mechanisms linking sedentary behavior with cognitive function are poorly understood. Previous studies have suggested that less sedentary time (or more physical activity) is associated with fewer brain lesions such as brain atrophy and white matter hyperintensities (WMHs) [15, 16]. In addition, the extent of brain atrophy and WMHs has been associated with accelerated cognitive impairment and dementia in middle-aged and older adults [17, 18]. Therefore, a conceivable hypothesis could be that structural brain properties may mediate, at least partly, the link of sedentary behavior with cognitive phenotypes.

In this community-based study of Chinese rural older adults, we sought to examine the dose-response associations of objectively measured total sedentary time and mean sedentary bout duration with global and domain-specific cognitive function, and further to explore the extent to which their associations with cognition are mediated by structural brain markers. We hypothesize that sedentary parameters are associated with poor cognitive outcomes, and that structural brain markers might partially mediate these associations.

## METHODS

### Participants

This population-based cross-sectional study used data from the baseline survey of the randomized controlled Multimodal INterventions to delay Dementia and disability in rural China (MIND-China), as previously described [19]. In August 2018-December 2020, a cluster (village)-based subsample (*n* = 2382) who were free of dementia and major mental health problems derived from the participants of MIND-China took part in the ActiGraph substudy [20]. Of these, 363 persons were excluded due to < 3 valid days of wearing the accelerometer (*n* = 281) and missing cognitive data (*n* = 82), leaving 2019 persons for the analysis. Of these, structural brain MRI data were available in 1009 participants. Supplementary Figure 1 shows the flowchart of study participants.

The Ethics Committee of Shandong Provincial Hospital approved the MIND-China protocol. Written informed consent was obtained from all participants. MIND-China was registered in the Chinese Clinical Trial Registry (registration no.: ChiCTR1800017758).

### Data collection and definitions

Data on demographics, lifestyles, medical history (e.g., coronary heart disease, diabetes, and stroke), and use of medications were collected through the face-to-face interview, neuropsychological testing, clinical examination, and laboratory tests [19]. Alcohol drinking and smoking status were categorized as never, former, and current drinking or smoking. Body mass index (BMI) and arterial blood pressure were measured as previously described [19]. Hypertension, diabetes, and dyslipidemia was defined as previously described [21]. The *APOE* genotype was measured using multiple-polymerase chain reaction amplification and was divided into any ɛ4 allele versus no ɛ4 allele [22].

### ActiGraph data collection and processing

Participants wore a triaxial accelerometer ActiGraph wGT3X-BT (ActiGraph, LLC, Pensacola, FL) on their hip, to affix to an elastic belt during all waking hours for 7 consecutive days, and only to remove it for swimming or bathing [20]. Wear season, accelerometer parameters, and non-wear time were defined as previously described [20]. We used data from participants who wore the device for≥10 h/day for≥3 days [23]. We defined sedentary behavior, light-intensity physical activity (LPA), and moderate-to-vigorous-intensity physical activity (MVPA) as counts per minute of 0–99, 100–1951, and≥1952, respectively [24].

### Definitions of sedentary behavior parameters

All sedentary accumulated metrics were assessed based on the most frequently used measure of sedentary time, defined as having < 100 counts per minute recorded on the vertical axis [20]. Total sedentary time was defined as the average time (minutes) per day spent in sedentary bout for≥1 min. A sedentary bout was defined as consecutive time (minutes) recorded on the accelerometer in which counts per minute were < 100. Mean sedentary bout duration was calculated using data from all valid days by dividing total sedentary time (minutes) by the total number of sedentary bouts. Lower bout durations indicate interrupted patterns, whereas higher bout durations indicate more accumulation patterns. We also assessed the prolonged sedentary time (time spent in sedentary bouts≥30 min) and the number of breaks per sedentary hour (a break was defined as≥1 min of non-sedentary bout after a sedentary bout).

### Neuropsychological test

The neuropsychological test battery was used to evaluate memory, attention, verbal fluency, and executive function [19, 25]. Specifically, memory was assessed with the Auditory Verbal Learning Test that included learning, delayed recall, and delayed recognition. Attention was assessed with the Trail Making Test-A and the sum of the Digit Span Forward. Verbal fluency was measured with the Verbal Fluency Test, including fruit, animal, and vegetable categories. Executive function was assessed with the Trail Making Test-B and Digit Span Backwards. The composite *z*-score for a specific cognitive domain was calculated by averaging the *z-*scores of relevant neuropsychological tests within the domain [26]. The composite *z*-score for global cognitive function was estimated by averaging the composite *z-*scores across the four cognitive domains.

### MRI acquisition and data processing

All eligible participants were scanned on either the Philips Archiva 3.0T MR System in the Liaocheng People’s Hospital (*n* = 95) or the Philips Ingenia 3.0T MR System in the Southwestern Lu Hospital (*n* = 914) [19].

For image processing, 3D T1-weighted images was used to quantify the brain volumes. We segmented brain volumes using the Computational Anatomy Toolbox in Matlab (http://dbm.neuro.uni-jena.de/cat12/) [27]. The Computational Anatomy Toolbox used a standard brain template of Montreal Neurological Institute to automatically normalize the skull-stripping brain, estimate the total intracranial volumes (TIV), and segment brain tissues into gray matter and white matter based on the Adaptive Maximum A Posteriori technique [28, 29]. Then, we extracted the bilateral hippocampal gray matter volumes using the Hammers atlas [30]. In addition, the T1 and T2-FLAIR images were processed in AccuBrain^®^ (BrainNow Medical Technology Ltd., Shenzhen, Guangdong, China) to acquire the volume of ventricles and WMH, respectively, as previously reported [30, 31]. In brief, AccuBrain^®^ automatically estimated the TIV using the normalized T1-weighted images. Subsequently, AccuBrain^®^ used the pre-defined normalized brain mask to segment brain tissue on T1-weighted image and then automatically estimated the ventricular volumes. Besides, AccuBrain^®^ used T2-FLAIR images to calculate WMH as previously described [32]. All segmentations were visually checked for accuracy, and segmentation failures were excluded from analysis or corrected if possible. WMH volume was log transformed owing to skewed distribution [32].

### Statistical analysis

General linear models were used to analyze the associations of sedentary parameters with cognitive z-scores. We reported the main results from three models: Model 1 was adjusted for demographics, *APOE* ɛ4 allele, MRI scanner, and accelerometer wear-season; Model 2 was additionally adjusted for lifestyle, cardiometabolic factors, coronary heart disease, and stroke; and MVPA was added in Model 3. The mediation role was assessed using the bootstrapping regression models adjusting for demographics and TIV. To account for correlation between total sedentary time and accelerometer wear-time, we standardized total sedentary time to 16 h of wear time per day using the residuals methods [33, 34].

The dose-response associations of cognitive *z-*scores with total sedentary time and mean sedentary bout duration were examined using restricted cubic spline functions [35]. We reported the results from models using 3 knots (10th, 50th, and 90th percentiles) for total sedentary time and 4 knots (5th, 35th, 65th, and 95th percentiles) for mean sedentary bout duration. The spline models were used to describe the nonlinear associations, and when the association was linear, the general linear model was used.

We used compositional isotemporal substitution model to estimate the associations with cognitive *z-*scores of replacing sedentary behavior variable with physical activity variable [36]. This model was performed using the R packages “Compositions”, “robCompositions”, and “lmtest”. Briefly, the compositional averages of daily sedentary behavior, LPA, and MVPA across the recording period were normalized to the proportion of 960 min (16 h). We examined how replacing 1 to 60 min of total sedentary time with other types of behavior was associated with cognitive *z-*scores. We estimated replacements up to 60 min to align with previous studies of mental health that used the compositional methods [37]. All the compositional isotemporal substitution models were adjusted for age, sex, education, *APOE* ɛ4 allele, accelerometer wear-season, smoking, alcohol consumption, BMI, stroke, coronary heart disease, diabetes, hypertension, and dyslipidemia.

Stata Statistical Software: Release 15 (StataCorp LLC., College Station, TX, USA) or R (R Foundation for Statistical Computing; Vienna, Austria) or IBM SPSS version 26.0 for Windows (IBM SPSS Inc., Chicago, IL, USA) was used for all analyses.

## RESULTS

### 3.1 Characteristics of study participants

Of the 2019 participants, the mean age was 69.9 (SD = 4.7) years, 58.9% were women, and 36.9% had no formal schooling (Table 1). Overall, participants spent 58.4% of daily waking time in sedentary behavior, 40.9% in LPA, and only 1.5% in MVPA.

**Table 1:** Characteristic of study participants

Characteristics	Total sample
	(*n* = 2,019)
Age (y)	69.9 (4.7)
Sex (female), *n* (%)	1190 (58.9)
Education, *n* (%)
No formal schooling	746 (36.9)
Primary school	881 (43.6)
Middle school or above	392 (19.4)
Current smoking, *n* (%)	390 (19.3)
Alcohol assumption, *n* (%)
Never	1,233 (61.1)
Former	160 (7.9)
Current	626 (31.0)
Body mass index (kg/m^2^)	25.1 (3.7)
Hypertension, *n* (%)	1,375 (68.1)
Diabetes, *n* (%)	305 (15.1)
Dyslipidemia, *n* (%)	464 (23.0)
Stroke, *n* (%)	274 (13.6)
Coronary heart disease, *n* (%)	434 (21.5)
*APOE* ɛ4 allele, *n* (%)	294 (14.6)
Daily wear time, min	846.9 (80.8)
% in Sedentary behavior	58.4 (13.4)
% in LPA	40.2 (12.5)
% in MVPA	1.5 (1.9)
Mean sedentary bout duration, minutes/bout	11.9 (6.5)

Data are mean (standard deviation), unless otherwise specified. SD, standard deviation; MRI, magnetic resonance imaging; *APOE*, apolipoprotein E gene; LPA, light-intensity physical activity; MVPA, moderate-to-vigorous-intensity physical activity.

### Sedentary behavior and cognitive function

**Fig. 1:**
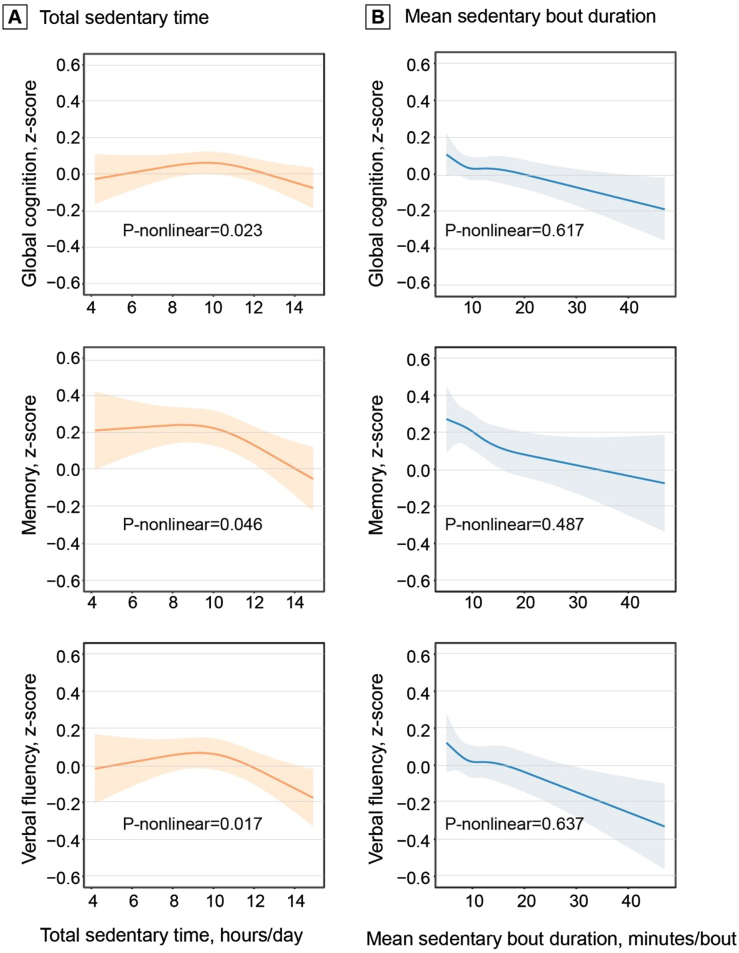
Dose-response associations of total sedentary time (A) and mean sedentary bout duration (B) with cognition. Data on the associations of total sedentary time and mean sedentary bout duration with global cognition, memory, and verbal fluency z-scores were fitted by restricted cubic spline models. The solid lines and shaded areas represent the β coefficients and 95% confidence intervals, respectively. Models were adjusted for age, sex, education, *APOE* ɛ4 allele, accelerometer wear-season, smoking, alcohol consumption, body mass index, stroke, coronary heart disease, diabetes, hypertension, dyslipidemia, and moderate-to-vigorous physical activity. Total sedentary time was corrected for accelerometer wear-time and expressed as the estimated sedentary time (hours) per day given a standardized 16 hours of wearing accelerometer.

In the ActiGraph sample (*n* = 2,019), adjusting for demographics, *APOE* ɛ4, and accelerometer wear-reason, greater total sedentary time was significantly associated with lower *z-*scores of memory and verbal fluency, but not with global cognition, attention, and executive function. The associations remained significant after adjusting for additional covariates and MVPA (Table 2). Restricted cubic spline regression analysis suggested non-linear relationships of total sedentary time with global cognition, memory, and verbal fluency z-scores (all *p*-nonlinear < 0.05, Fig. 1A). The restricted cubic splines showed a linear association of total sedentary time with the cognitive z-scores after certain thresholds of total sedentary time. In addition, longer mean sedentary bout duration was linearly associated with lower *z-*scores of global cognition, memory, and verbal fluency (all *p*-nonlinear > 0.05, Fig. 1B).

**Table 2:** Association of sedentary parameters with cognitive function (n = 2019)

Cognitive function (outcomes)	β coefficient (95% confidence interval), cognitive z-score		
	Model 1^b^	Model 2^b^	Model 3^b^
Global cognition
Total sedentary time^a^	–0.011 (–0.023, 0.001)	–0.008 (–0.021, 0.004)	–0.008 (–0.022, 0.006)
Mean sedentary bout duration	–0.007 (–0.011, –0.003)^‡^	–0.006 (–0.010, –0.002)^†^	–0.006 (–0.010, –0.002)^†^
Memory
Total sedentary time^a^	–0.034 (–0.053, –0.016)^‡^	–0.029 (–0.047, –0.010)^†^	–0.029 (–0.050, –0.007)^†^
Mean sedentary bout duration	–0.012 (–0.018, –0.006)^‡^	–0.009 (–0.016, –0.003)^†^	–0.009 (–0.015, –0.003)^†^
Verbal fluency
Total sedentary time^a^	–0.022 (–0.038, –0.005)^†^	–0.018 (–0.035, –0.002)*	–0.019 (–0.038, –0.000)*
Mean sedentary bout duration	–0.011 (–0.016, –0.005)^‡^	–0.009 (–0.015, –0.004)^‡^	–0.009 (–0.015, –0.004)^†^
Attention
Total sedentary time^a^	0.008 (–0.008, 0.025)	0.010 (–0.006, 0.027)	0.010 (–0.009, 0.029)
Mean sedentary bout duration	–0.004 (–0.009, 0.001)	–0.003 (–0.008, 0.003)	–0.003 (–0.009, 0.002)
Executive function
Total sedentary time^a^	0.004 (–0.013, 0.020)	0.003 (–0.013, 0.020)	0.007 (–0.012, 0.026)
Mean sedentary bout duration	–0.003 (–0.008, 0.002)	–0.002 (–0.008, 0.003)	–0.002 (–0.007, 0.004)

^a^Total sedentary time was corrected for accelerometer wear-time and expressed as the estimated sedentary time (hours) per day given a standardized 16 hours of wearing accelerometer. ^b^Model 1 was adjusted for age, sex, education, *APOE* ɛ4 allele, and accelerometer wear-season; Model 2 was additionally adjusted for smoking, alcohol consumption, body mass index, stroke, coronary heart disease, diabetes, hypertension, and dyslipidemia; and in Model 3 moderate-to-vigorous physical activity was added to model 2. ^*^p < 0.05, ^†^*p* < 0.01, ^‡^*p* < 0.001.

**Fig. 2:**
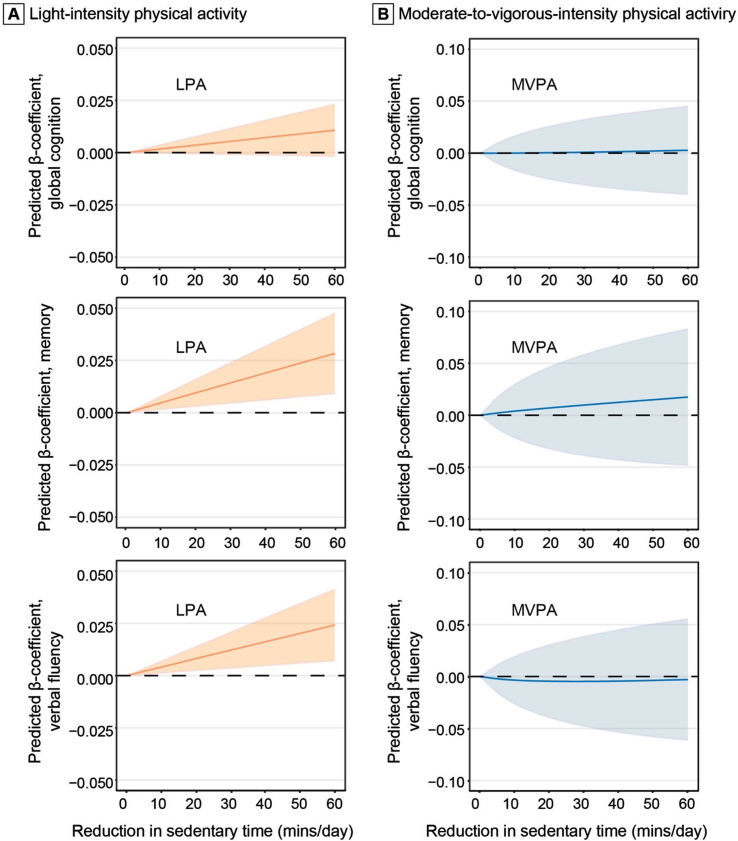
Associations of replacing daily sedentary time with light-intensity physical activity (A) and moderate-to-vagarous-intensity physical activity (B) with cognition (*n* = 2,019). The predicted β-coefficients and 95% confidence intervals of global cognition, memory, and verbal fluency z-scores when reallocating a given amount of time between daily sedentary time and physical activity while keeping the remaining components constant as compositional means in 16 hours (we omitted 8 hours of sleep time per day). The results were controlled for age, sex, education, *APOE* ɛ4 allele, accelerometer wear-season, smoking, alcohol consumption, body mass index, stroke, coronary heart disease, diabetes, hypertension, and dyslipidemia. LPA, light-intensity physical activity; MVPA, moderate-to-vigorous-intensity physical activity.

In addition, we examined the potential effect on the association with cognitive z-score of replacing 1-60 min of sedentary behavior with an equal amount of LPA and MVPA in the total sample (Fig. 2). Replacing 60 min of sedentary behavior with 60 min of LPA in a 16-h period was associated with higher *z-*scores of memory (β= 0.0283; 95% CI: 0.0091–0.0476) and verbal fluency (0.0242; 0.0070-0.0413). However, replacing sedentary time with MVPA was not significantly associated with memory and verbal fluency *z-*scores.

### Mediating effect of brain markers on associations of sedentary behavior with cognition

In the MRI sample (*n* = 1,009), controlling for demographics and TIV, mean sedentary bout duration was significantly associated with a lower global cognitive z-score and was marginally associated with a lower verbal fluency z-score (*p* = 0.074), but not with the memory z-score (Table 3). When volumes of WMH, total white matter, ventricles, and hippocampus were entered into the mediation model separately, the associations of mean sedentary bout duration with global cognition, memory, and verbal fluency *z-*scores were significantly attenuated, and the mediation in the associations with global cognition and fluency z-scores was statistically significant (Table 3). The volumes of WMH, ventricles, and hippocampus, but not total white matter, significantly mediated the associations of mean sedentary bout duration with memory z-score. When entering the four structural brain MRI markers into the mediation models separately, the direct association of mean bout duration with global cognition and fluency z-scores became statistically nonsignificant (Table 3).

**Table 3:** Mediating effects of structural brain markers on the associations of mean sedentary bout duration with cognitive function (*n* = 1009)

Mean sedentary bout duration^b^	β coefficient (95% confidence interval)^a^		
and mediators	Global cognition, z-score	Memory, z-score	Verbal fluency, z-score
Total effect of sedentary behavior	–0.0068 (–0.0134, –0.0002)	–0.0078 (–0.0177, 0.0020)	–0.0081 (–0.0169, 0.0008)
Mediator, WMH volume
Direct effect of sedentary behavior	–0.0046 (–0.0112, 0.0020)	–0.0048 (–0.0147, 0.0050)	–0.0058 (–0.0147, 0.0031)
Mediating effect	–0.0022 (–0.0040, –0.0011)	–0.0030 (–0.0054, –0.0014)	–0.0022 (–0.0043, –0.0009)
Percent mediation	32.35%	38.46%	27.16%
Mediator, total white matter volume
Direct effect of sedentary behavior	–0.0053 (–0.0120, 0.0013)	–0.0069 (–0.0168, 0.0031)	–0.0057 (–0.0147, 0.0032)
Mediating effect	–0.0015 (–0.0027, –0.0005)	–0.0010 (–0.0027, 0.0005)	–0.0023 (–0.0040, –0.0009)
Percent mediation	22.06%	12.82%	28.40%
Mediator, ventricular volume
Direct effect of sedentary behavior	–0.0047 (–0.0114, 0.0020)	–0.0058 (–0.0158, 0.0042)	–0.0062 (–0.0151, 0.0028)
Mediating effect	–0.0021 (–0.0035, –0.0009)	–0.0020 (–0.0041, –0.0003)	–0.0019 (–0.0037, –0.0005)
Percent mediation	30.88%	25.64%	23.46%
Mediator, hippocampal volume
Direct effect of sedentary behavior	–0.0052 (–0.0118, 0.0014)	–0.0058 (–0.0156, 0.0041)	–0.0063 (–0.0152, 0.0026)
Mediating effect	–0.0016 (–0.0031, –0.0004)	–0.0020 (–0.0041, –0.0005)	–0.0017 (–0.0036, –0.0004)
Percent mediation	23.53%	25.64%	20.99%

WMH, white matter hyperintensity. ^a^ β coefficient (95% confidence interval) was derived from the mediation models, controlling for age, sex, and education. ^b^Mean sedentary bout duration was calculated as total sedentary time divided by total number of sedentary bouts.

## DISCUSSION

This population-based study engaged older adults who were living in rural communities in China. The main findings from this study were summarized as follows. First, there was a dose-response association of longer mean sedentary bout duration (or less frequent breaks in sedentary time) with lower *z-*scores of global cognition, verbal fluency, and memory independent of MVPA, whereas the linear association of greater sedentary time with poorer global cognitive function, verbal fluency, and memory existed only among older people with long sedentary time (> 10 h/day); Second, breaking up sedentary time with LPA was associated with better verbal fluency and memory function; Finally, the associations of mean bout duration with global cognition, memory, and verbal fluency were largely mediated by WMH, white matter, ventricular, and hippocampal volumes.

The pooling data of five population-based cohorts showed neither cross-sectional nor longitudinal association between self-reported total undifferentiated sedentary time and global cognition [11]. The cross-sectional data from the UK Biobank showed associations of more time spent watching TV with poor cognitive function [38]. However, studies based solely on self-reported sedentary time are prone to recall bias towards underreporting the sedentary time [6, 7], which may underestimate its association with health outcomes. Our study provided solid evidence supporting an association between accelerometer-assessed sedentary time and poor cognitive function in multiple domains among rural-dwelling Chinese older adults.

Notably, our analysis revealed a dose-response relationship between daily prolonged sedentary time and poor cognitive function, especially among individuals with long sedentary time (> 10 h/day). Furthermore, we found that replacing sedentary time with equal amount of LPA might help maintain memory and fluency function in older adults, which is consistent with the findings from the cross-sectional Arakawa 85 + study in Japan [39]. Collectively, these findings imply that movement *per se* (e.g., sitting less and more frequently taking breaks during sedentary time), rather than intensity, is crucial for cognitive health in older adults. From the public health perspective, this has significant implications for promoting brain health in old age.

Another key contribution of our study was that apart from total sedentary time, the patterns of sedentary behavior were important for brain health and cognition. Previous studies have repeatedly linked prolonged sedentary time (a metric that is highly correlated with longer sedentary bout duration and fewer breaks in sedentary behavior) with cardiometabolic risk factors, cardiovascular disease, and all-cause mortality [33, 40, 41]. These findings highlight the relevance of “prolonger” versus “breaker” hypothesis, i.e., both the amount of sedentary time and the manner in which it is accumulated are important for brain and cognitive health. Our study revealed that prolonged uninterrupted sedentary time was associated with poor memory and verbal fluency function, while more frequent breaks in sedentary behavior with LPA were associated with better memory and verbal fluency.

Clarifying the mediations of structural brain markers in the associations of sedentary behavior with cognition represents the third important contribution of our study. The association between structural brain measures and cognition has been well established [17, 18]. In addition, previous studies have frequently linked sedentary time with structural brain markers in older adults. For example, the cross-sectional data from the Icelandic AGES-Reykjavik Study showed that self-reported physical activity was associated with greater gray matter and white matter volumes [42]. We further revealed that the association of prolonged uninterrupted sedentary behavior with global and domain-specific cognition was largely mediated by MRI markers of WMH and brain atrophy. The community-based studies of older adults in the USA and Sweden showed that larger WMH volume was associated with reduced volumes of cortical gray matter and the hippocampus [43–45]. Our study supported the chain mediating role of both WMH and hippocampal volumes in the association between long sedentary bout duration (or less frequent breaks in sedentary behavior or prolonged sedentary time) and poor cognition. Taken together, these studies support the view that prolonged, uninterrupted sedentary time could be linked with poor cognitive function through mixed pathologies of cerebral microvascular lesions and neurodegeneration.

Several pathophysiological pathways may link sedentary time and patterns with structural brain alterations and poor cognition in older adults. First, prolonged uninterrupted sedentary behavior could affect brain glucose metabolism *via* elevating postprandial hyperglycemia, proportional hyperinsulinemia, and subsequent insulin resistance [46, 47], thus affecting cognitive function. Furthermore, prolonged sedentary time was associated with reduced shear stress in lower limb blood vessels [48], resulting in viscous blood flow in the lower limb and lower cerebral blood flow [49], which in turn may damage brain structure and lead to cognitive dysfunction [50].

The major strength of our study refers to the community-based design that engaged the largely ignored demographic group of rural-dwelling older adults in China by the research community, in which the objectively measured data on sedentary parameters were integrated with comprehensive cognitive and structural brain MRI data. Thus, we were able to explore not only the relationship of sedentary parameters with cognitive function but also the potential neuropathological pathways underlying the relationships. However, some limitations of our study deserve mentioning. First, the cross-sectional nature of the study design prevented us from making causal inferences or interpretations for any of the observed associations and mediations, and the cross-sectional association was subject to selective survival bias that usually led to an underestimation of the true associations. Furthermore, there was a time gap between assessments of cognitive function (March-September 2018) and collection of ActiGraph and brain MRI data (August 2018-December 2020) that should be considered when interpreting the results. Third, the ActiGraph accelerometer cannot distinguish different postures (e.g., sitting and standing), thus sedentary time may be overestimated by including time of certain standing positions with no movement. Finally, the study sample was derived from only one rural area in western Shandong Province and may not be representative of older population in rural China, which should be kept in mind when generalizing our study findings to other rural populations.

In conclusion, this population-based study of rural-dwelling older adults clearly revealed that accumulation patterns of prolonged sedentary time were linearly associated with poor global cognition, memory, and verbal fluency function, in which the association was largely mediated by structural brain lesions. Furthermore, breaking up sedentary time with LPA was associated with better memory and verbal fluency function. Future longitudinal studies will help further increase our understanding of the causal relationship and potential mechanisms linking sedentary behavior with structural brain aging and cognitive dysfunction in older people. This is highly relevant for the development of interventions to achieve healthy brain aging in rural populations.

## Supplementary Material

Supplementary Material

## Data Availability

The datasets used and/or analyzed during the current study are available from the corresponding author upon reasonable request.
