# Supplementary material for: Accelerometer-Measured Sedentary Behavior Patterns, Brain Structure, and Cognitive Function in Dementia-Free Older Adults: A Population-Based Study
Source: J Alzheimers Dis. 2023 Nov 7;96(2):657–68. doi: 10.3233/JAD-230575 (PMC10657675; doi:10.3233/JAD-230575)
Supplement: Supplementary Material [file jad-96-jad230575-s001.pdf]

Supplementary Material

Accelerometer-Measured Sedentary Behavior Patterns, Brain Structure, and Cognitive Function in Dementia-Free Older Adults: A Population-Based Study

Supplementary Figure 1. Flowchart of study participants

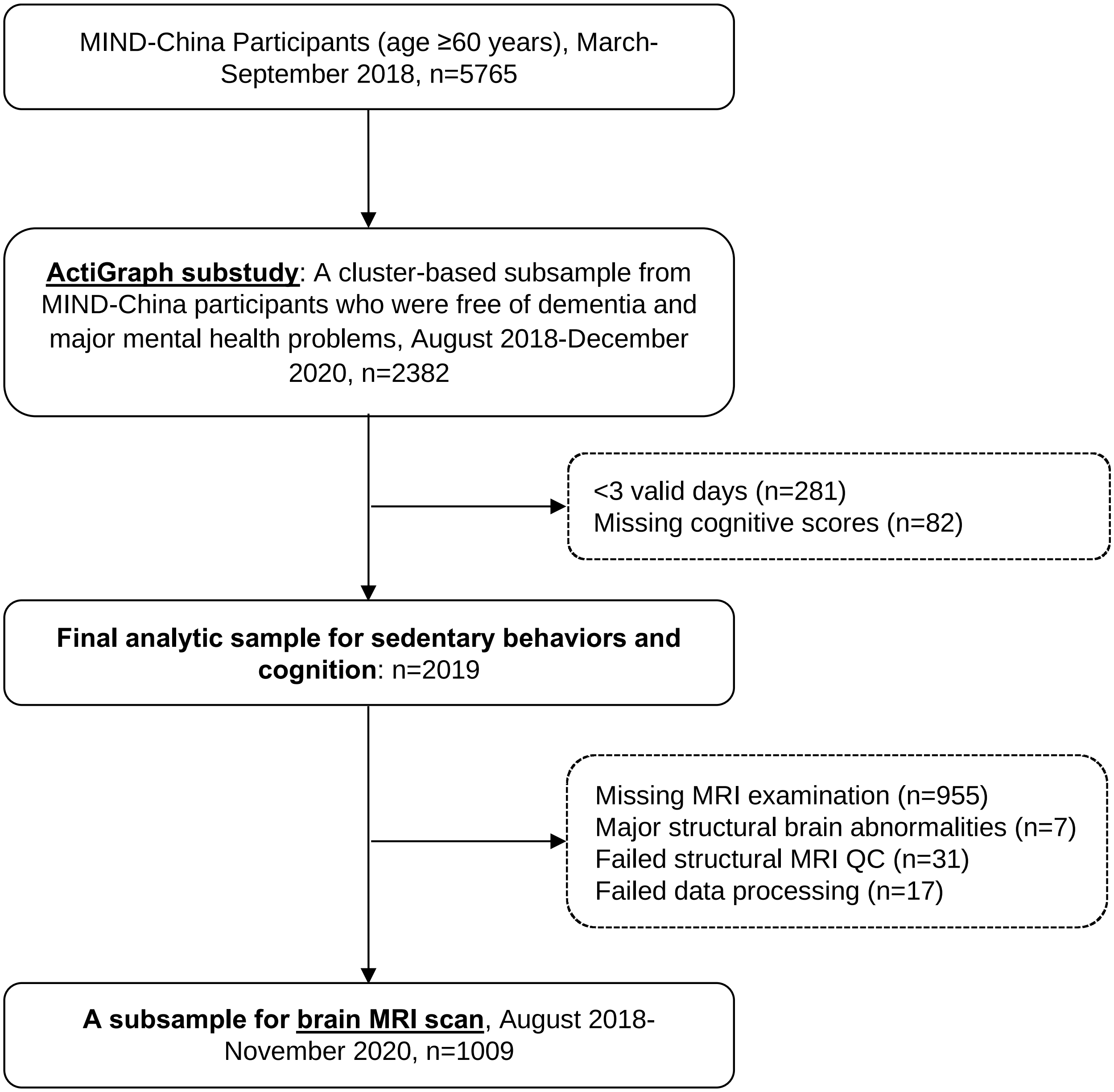

MIND-China, multimodal interventions to delay dementia and disability in rural China; MRI, magnetic resonance imaging; QC, quality control.
